# Retrospective analysis of hospitalization costs using two payment systems: the diagnosis related groups (DRG) and the Queralt system, a newly developed case-mix tool for hospitalized patients

**DOI:** 10.1186/s13561-024-00522-6

**Published:** 2024-06-26

**Authors:** Júlia Folguera, Elisabet Buj, David Monterde, Gerard Carot-Sans, Isaac Cano, Jordi Piera-Jiménez, Miquel Arrufat

**Affiliations:** 1 Catalan Health Service, Gran Via de les Corts Catalanes 587, Barcelona, 08007 Spain; 2 Digitalization for the Sustainability of the Healthcare System (DS3) – Institut d’Investigacions Biomèdiques de Bellvitge (IDIBELL), Barcelona, Spain; 3 Catalan Institute of Health, Barcelona, Spain; 4 Fundació de Recerca Clinic Barcelona - Institut d’Investigacions Biomèdiques August Pi i Sunyer (FRCB- IDIBAPS), Universitat de Barcelona, Barcelona, Spain; 5 Faculty of Informatics, Telecommunications and Multimedia, Universitat Oberta de Catalunya, Barcelona, Spain

**Keywords:** Hospital costs, Case-mix tools, Diagnosis-related groups, Queralt system

## Abstract

**Background:**

Hospital services are typically reimbursed using case-mix tools that group patients according to diagnoses and procedures. We recently developed a case-mix tool (i.e., the Queralt system) aimed at supporting clinicians in patient management. In this study, we compared the performance of a broadly used tool (i.e., the APR-DRG) with the Queralt system.

**Methods:**

Retrospective analysis of all admissions occurred in any of the eight hospitals of the Catalan Institute of Health (i.e., approximately, 30% of all hospitalizations in Catalonia) during 2019. Costs were retrieved from a full cost accounting. Electronic health records were used to calculate the APR-DRG group and the Queralt index, and its different sub-indices for diagnoses (main diagnosis, comorbidities on admission, andcomplications occurred during hospital stay) and procedures (main and secondary procedures). The primary objective was the predictive capacity of the tools; we also investigated efficiency and within-group homogeneity.

**Results:**

The analysis included 166,837 hospitalization episodes, with a mean cost of € 4,935 (median 2,616; interquartile range 1,011–5,543). The components of the Queralt system had higher efficiency (i.e., the percentage of costs and hospitalizations covered by increasing percentages of groups from each case-mix tool) and lower heterogeneity. The logistic model for predicting costs at pre-stablished thresholds (i.e., 80th, 90th, and 95th percentiles) showed better performance for the Queralt system, particularly when combining diagnoses and procedures (DP): the area under the receiver operating characteristics curve for the 80th, 90th, 95th cost percentiles were 0.904, 0.882, and 0.863 for the APR-DRG, and 0.958, 0.945, and 0.928 for the Queralt DP; the corresponding values of area under the precision-recall curve were 0.522, 0.604, and 0.699 for the APR-DRG, and 0.748, 0.7966, and 0.834 for the Queralt DP. Likewise, the linear model for predicting the actual cost fitted better in the case of the Queralt system.

**Conclusions:**

The Queralt system, originally developed to predict hospital outcomes, has good performance and efficiency for predicting hospitalization costs.

**Supplementary Information:**

The online version contains supplementary material available at 10.1186/s13561-024-00522-6.

## Background

The increasing complexity of healthcare systems and the diversity of healthcare services experienced in the last decades have transformed how healthcare providers are paid for their services [1]. This interest has been particularly important in the hospital setting, which is the most costly of the healthcare system [2].

Traditional payment approaches, such as retrospective fee-for-service models —where providers are paid for the services and procedures delivered—, have discouraged efficiency and quality, often leading to unnecessary procedures and longer average values of the length of stay. Similarly, prospective per-diem payments – where providers are paid for a type of in-patient day, hampered healthcare planning and budgeting [3]. To overcome these drawbacks, many healthcare systems worldwide initiated a transition towards case-mix tools, which summarize a large number of items related to patient complexity and procedures into a limited number of groups with certain characteristics in common, allowing classification of all hospitalizations.

Case-mix tools for summarizing the clinical complexity of patients have been extensively used in healthcare. However, most assessments reported in the literature have investigated clinical or hard endpoints, uch as mortality (in-hospital and at different time points after discharge), while the performance of these tools for cost estimate have been scarce [4]. More than 20 years ago, Ash et al. showed how diagnoses and diagnostic groups in general aligned with the actual costs for procedure [5]. Similarly, Huang et al. compared the ability of several case-mix tools developed for clinical purposes to inform healthcare costs. The authors found that, although all case-mix tools performed acceptably for predicting costs, proprietary tools were better, limiting their adaptability to specific environments [6]. Finally, it is worth mentioning that, recently, with the advent of machine learning use in healthcare, some authors have improved cost predictions by including information on social determinants [7].

The most popular case-mix tool is the diagnosis-related groups (DRG), which rapidly expanded across the globe. The DRG was created at Yale University in the 1960s to define the services provided by each hospital and manage costs and quality and has rapidly been implemented across the globe. In its more recent version, all-patient refined (APR-DRG), it has several advantages, including transparency, exhaustivity (e.g., accounts for severity and risk levels), and the possibility of measuring quality indicators across hospitals and even countries [8]. However, it is costly and inflexible: it does not use all the administrative data available in each healthcare system and it is usually trained with the USA population and not the local one, thus precluding healthcare systems from tailoring this tool to their needs and capabilities in terms of data collection. Furthermore, the APR-DRG accounts for diagnoses and procedures but not complications that may occur during hospitalization and may impact the overall cost.

Recently, we developed a comprehensive risk adjustment tool for predicting health outcomes in hospitalized patients: the Queralt system [9]. The tool was designed as a family of indices that summarize the principal diagnosis (Queralt DxP), in-hospital complications (Queralt DxC), and diagnoses on admission (Queralt DxS) based on weighted sums of over 2,100 diagnostic codes. All indices can be summarized in the Queralt Dx. The Queralt System has shown a high capacity to predict critical events such as death or admission to the intensive care unit (ICU) in all-cause hospitalizations [9] and disease-specific hospitalizations [10]. Additionally, the Queralt System includes two indices for primary and secondary procedures, respectively. Like in the case of diagnoses, the procedure indices can be combined in a single one (i.e., Queralt Px). However, its performance as a paying system for hospital services has not been assessed. In this retrospective analysis of costs, we compared the performance of the APR-DRG and the Queralt Dx and Px indices for estimating the costs of hospital services.

## Methods

### Study design and setting

This was a cost analysis of hospital services that included all hospitals in the Catalan Institute of Health (ICS for Catalan *Institut Català de la Salut*). The ICS is the leading healthcare provider in Catalonia (North-East Spain), and delivers public and universal healthcare to its reference population, accounting for approximately 30% of all hospitalizations in Catalonia. Three of the eight hospitals managed by the ICS are tertiary hospitals with specialized reference services that receive referrals from other centers of the public healthcare network. Hospitals of the ICS are currently paid by the public healthcare insurer (i.e., the Catalan Health Service) on an activity-based payment system. Service costs are established based on full cost accounting, which considers direct and indirect costs of healthcare service delivery, aimed at establishing the cost of a given provision (i.e., the smallest unit of care received by the patient). The provision-based grouping allows estimating the cost per episode, patient, or diagnosis by grouping several provisions.

Two type of costs were considered: direct costs and indirect costs. Direct costs included all costs that the provider entity (i.e., the hospital) allocates to the units providing care activities (e.g., cardiology department, dermatology department…). These costs encompass personnel of the given unit, pharmacy, and supplies that are central for the healthcare activity. Considering that cost may vary according to services, a weight of relative unit of value is assigned to each unit. The cost for each service is added to each recorded patients within the hospitalization episode; the sum of all services represents the patient cost during the admission period.

Indirect costs include all attributed to units that do not perform healthcare activity per se. Examples of these units include admissions, clinical documentation, sterilization, and accounting. Indirect costs are distributed proportionally to healthcare units at the hospital level. Proportionality criteria are defined for the entire organization for each type of cost (e.g., cleaning expenses are assigned based on weighted square meters per unit type, management expenses based on staffing).

In this analysis, we retrospectively collected the costs of all regular hospitalizations (i.e., excluding hospitalizations because of major surgery and other hospital procedures) that occurred in any of the ICS hospitals between January 01 and December 31, 2019. Registries of DRG groups with less than 25 cases and those without grouping criteria in either the DRG or the Queralt system were removed. The cost estimate was conducted in euros for 2019.

Cost information was retrieved without personal or health information; therefore, the analysis did not meet the criteria for being considered a clinical study; therefore, approval by an independent ethics committee was deemed not necessary by the Bellvitge Independent Ethics Committee (Spain).

### Case-mix tools

The development of the Queralt system for diagnoses is described elsewhere [9]; the software and user’s manual of the Queralt system can be accessed from https://ics.gencat.cat/ca/assistencia/coneixement-assistencial/sistema-de-Queralt/. The Queralt system considers diagnoses and procedures separately, both summarized as indices (i.e., numerical values that measure the complexity of hospitalization). In the case of diagnoses, the system provides three sub-indices: the main diagnosis (QIDP), comorbidities present on admission (QIDA), and complications occurred during hospital stay (QIDC). The three indices regarding diagnoses can be summarized in the Queralt index for diagnoses (QID), which is the sum of QIDP + QIDA + QIDC. Similarly, the Queralt system has two sub-indices for procedures: main procedure (QIPP) and secondary procedures (QIPS). Like the diagnosis indices, procedures indices can be summarized in a Queralt indices for procedures (QIP), which is the sum of QIPP + QIPS. The distribution of each of the summary indices (i.e., QIP and QID) is used to define complexity groups for diagnoses (D_strata) and procedures (P_strata). The supplementary methods (Supplementary appendix) provides details regarding the definition of the complexity groups or strata. In this analysis, we used both the numerical indices (QID and QIP) and the groups or strata (D_strata and P_strata). We also combined the two last (i.e., scalar product of D_strata and P_strata) to obtain an exhaustive stratification based on both, diagnoses and procedures (DP_strata).

The DRG system is a for-profit case-mix tool designed for assessing patients’ complexity and service payment that allows assigning hospital services to comparable groups and determining the weight or price for each of the groups of products [11]. In Catalonia, the APR-DRG is used. The APR-DRG divides each base DRG into four risk levels (based on mortality risk) and four severity levels (based on the length-of-stay and other hospital outcomes). In this study, we used the DRG Severity (i.e., the Cartesian combination of the base DRG and severity levels related to resource utilization).

### Outcomes and statistical approach

The primary objective was to compare the predictive capacity of costs between the different case-mix tools. This objective was addressed using two outcomes: the cost as a numerical (continuous) variable in order to study if the groupers could predict the distribution of costs, and the cost (as categorical, binary) stratified by the 80th, 90th, and 95th percentiles to investigate the capacity to predict high costs.

Before model building, we examined the classification systems using two different indicators: efficiency and within-group homogeneity. The efficiency was defined as the percentage of costs and hospitalizations covered by increasing percentages of groups from each case-mix tool. The within-group homogeneity was assessed by estimating the coefficient of variation (i.e., standard deviation over mean) for costs in each group of the case-mix tool.

To test the predictive capacity of the case-mix tools, we built two model types for each grouper: D_strata, P_strata, and DRG severity. First, we performed a logistic regression for each grouper to predict high cost based on three thresholds of cost distribution: percentiles 80th, 90th, and 95th. Then, we conducted a lognormal regression for each grouper to predict the actual cost. Since risk and severity levels make sense only within a given DRG base group, we stratified all analyses for each DRG base group. DRG base groups with less than 25 registries were removed. The predictive validity of the models was assessed using the reduction in variance, as described elsewhere [12, 13]. Briefly, the reduction in variance indicates the amount of variation in the dependent variable explained by the classification of items. The performance of the logistic regression models for high costs was assessed using the receiving operating characteristics (ROC) and precision-recall (PR) curves. Additionally, we conducted linear models for descriptive purposes; the the performance of the linear models for actual costs was represented by plotting the expected and observed distribution of costs across groups.

## Results

### Overall costs

**Fig. 1:**
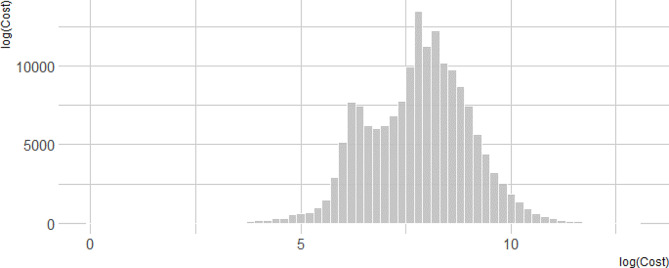
Distribution of cost (logarithm)

Our analysis included 166,837 hospitalization episodes, with a mean cost of € 4,935 (median 2,616; interquartile range 1,011–5,543). Table 1 summarizes the mean and median costs according to age groups and sex; the interactions between these demographic characteristics are provided in Table S1 (Supplementary Appendix). Overall, hospitalization costs within the investigated period amounted to 2,006 million euros; of them, 1,496 million were direct costs and 513 indirect costs. Figure 1 summarizes the distribution of costs per episode.

**Table 1:** Costs (in €) according to demographic characteristics

	Mean	Median (IQR)
Sex		
Male	5337.4	2671 (1003–6025)
Female	4509.9	2573 (1019–5122)
Age group		
0–9	3589.3	1652 (883–3208)
10–19	4537.5	2168 (967–4258)
20–29	4173.7	2503 (1213–4203)
30–39	4050.4	2548 (1414–4208.25)
40–49	5013.5	2749 (1081–5663)
50–59	5840.4	3139 (1178–6642)
60–69	6048.3	3241 (1220–7000)
70–79	5569.5	3032 (1074–6625)
80–89	4229.7	2374 (764–5169)
90–99	3006.2	1771 (617–4020)
100+	2782.9	1154 (526–4112)

IQR: interquartile range, defined by the 25th and 75th percentiles

Figure S1 shows the distribution of average cost and number of hospitalizations for twenty relevant DRG groups. The average cost according to sex and age for eight relevant causes of hospitalization (either diagnoses or procedures, with overlap between DRG and Queralt groups) is shown in Figure S2.

### Efficiency and heterogeneity

**Fig. 2:**
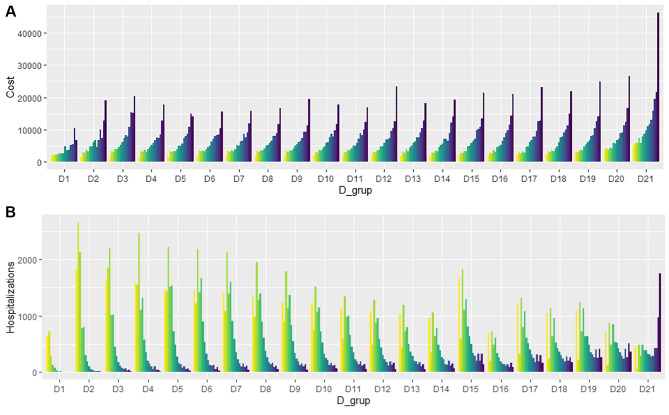
Distribution of costs (**A**) and hospitalizations (**B**) within each of the D_strata and, within them, P_strata. Colors show the relative values for the P_strata, from yellow (lowest values) to dark blue (highest values)

Figure 2 summarizes the values of costs (A) and frequency of hospitalization episodes (B) for each of the DP_strata (grouped first according to D_strata and, within them, the P_strata). The distribution of costs and hospitalizations revealed that the P_strata grouper explained better the differences in costs and episodes than the D_strata (Fig. 2). Also, we saw that less costly hospitalizations were remarkably more frequent than high-costly ones.

**Fig. 3:**
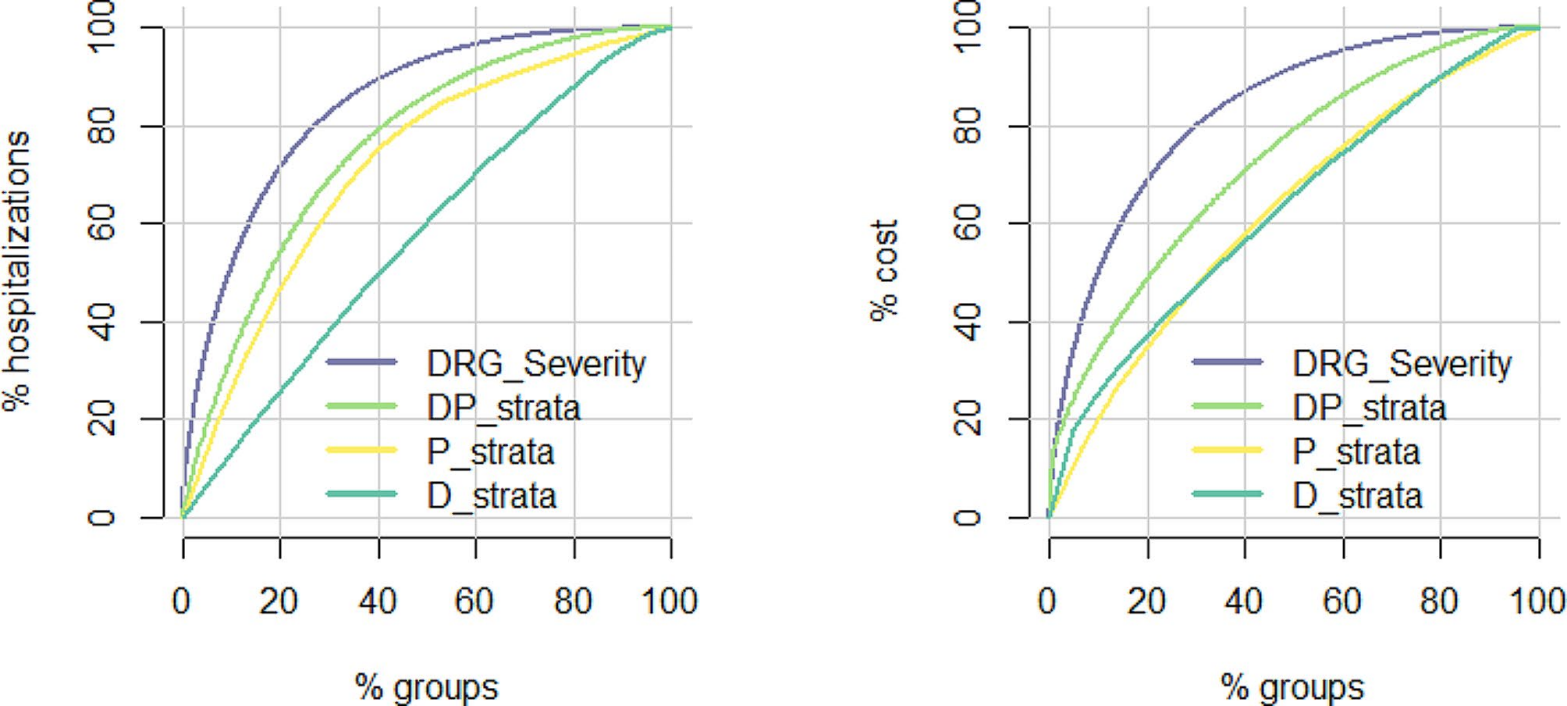
Efficiency analysis of the DRG and Queralt System components

Regarding efficiency, we also investigated the percentage of costs and hospitalizations covered by each percentage of the Queralt and DRG groups (Fig. 3). The D_strata of the Queralt System showed the highest efficiency for hospitalizations (i.e., closest to the center diagonal, indicating proportionality). The highest efficiency for costs was also observed for the P_strata and D_strata components. In the case of the DRG system, a moderate percentage of groups covered high percentages of costs and hospitalizations, and the coverage improved very little when increasing the number of groups, indicating the low efficiency of the system.

**Fig. 4:**
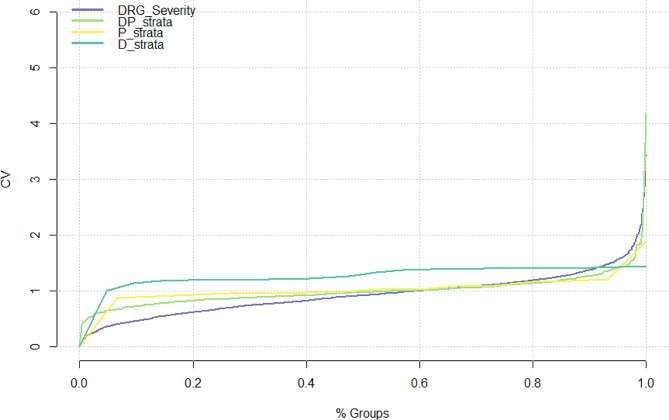
Coefficient of variation of groups from the different case-mix tools

The components of the Queralt system showed a relatively stable coefficient of variation around 1 across most groups. On the other hand, the DRG groups showed a more heterogeneous profile, with some groups associated with the lowest coefficient of variation and some others with the highest (Fig. 4).

### Predictive model

The reduction in variance of the predictive based on DRG_Severity grouper was 0.487. The corresponding values for the D_strata, P_strata, and DP_strata of the Queralt system were 0.149, 0.326, and 0.379, respectively, indicating that the DRG system (DRG_Severity) has slightly higher predictive validity.

**Fig. 5:**
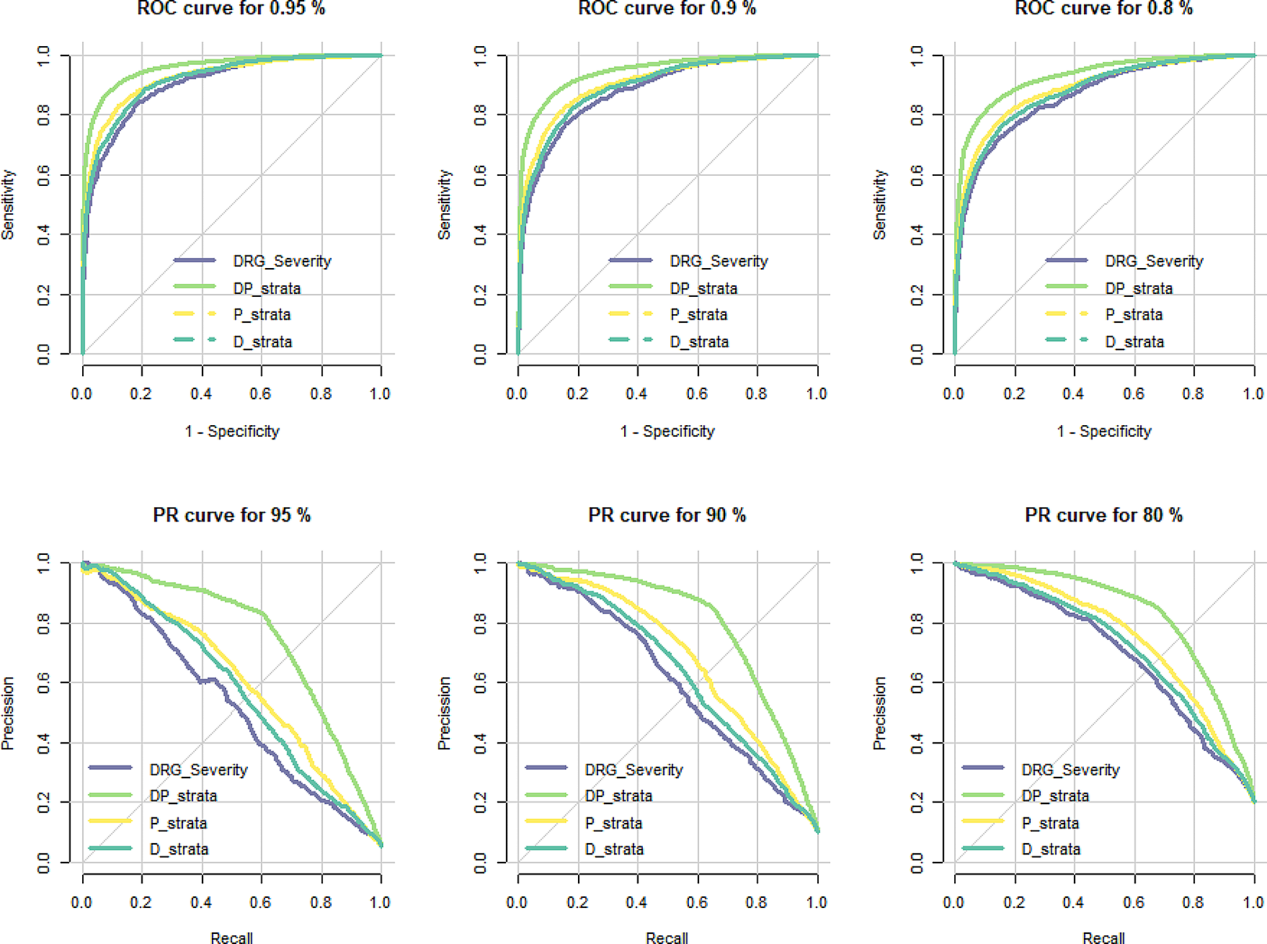
Performance of the logistic regression models with components from the DRG and Queralt systems for predicting high hospital costs when considering three percentile thresholds of the actual cost: 80th, 90th, and 95th. ROC: receiving operating characteristics. PR: precision-recall. The values for area under the curve are provided in Table S2 (Supplementary appendix)

According to the ROC analysis, the DP_strata of the Queralt system had the highest performance in predicting high hospital costs at all thresholds established (i.e., 80th, 90th, and 95th percentiles) (Fig. 5). The other components analyzed showed little differences. In the corresponding PR analysis, the superiority of the DP_Strata of the Queralt system was even more notorious, and the other two components of this system (i.e., the P_strata and D_strata) were slightly better than the DRG components.

**Fig. 6:**
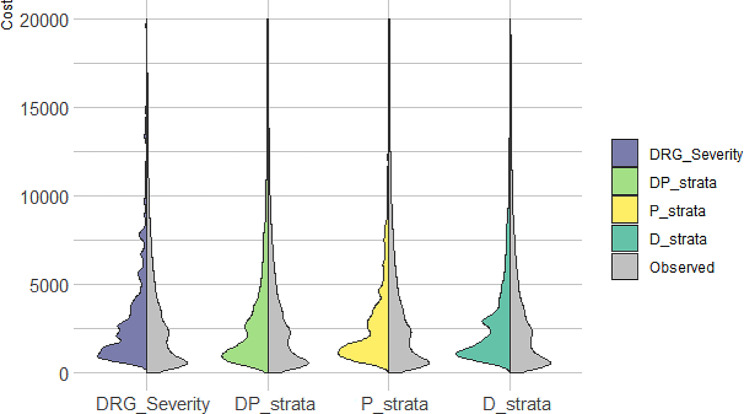
Distribution of the observed and expected cost according to linear models built based on each of the grouper system

Regarding the predictive capacity of the actual cost, the linear regression model based on the DP_strata of the Queralt system showed the closest distribution to the actual cost (Fig. 6).

## Discussion

### Summary of main findings

In this retrospective analysis of analytical costs in public hospitals in Catalonia, we found that a recently-developed case-mix tool for risk stratification in the hospital setting shows better performance (in terms of both efficiency and ability to predict costs) than the APR-DRG, a tool broadly used for reimbursement of hospital services.

### Diagnoses and procedures separately

Classical case-mix tools developed as an approach to reimbursement procedures, including but not limited to DRG, use pre-defined combinations of diagnoses and procedures to build risk groups. Alternatively, the Qurealt tool was conceived as a system of independent indices (including diagnoses and procedures) that can be used separately or combined, depending on the needs [9]. In our analysis, the distribution of costs and hospitalization events across diagnostic and procedure groups (D_strata and P_strata, respectively) revealed that procedures account for higher differences in the overall costs than diagnoses. This finding indicates that within a given diagnostic group, procedures may make an important difference in costs, which is consistent with cost analyses in specific settings that show how heterogeneous and difficult to predict may be the costs of hospital procedures despite sharing the same main diagnosis [14, 15]. Therefore, although the Queralt system ends up with a lower number of groups, the flexibility of combining them is likely to better describe the actual scenario of hospitalizations.

### The number of groups needed

Case mix tools for reimbursement face the challenge of finding the optimal balance in the trade-off between the number of groups and the efficiency of the tool (briefly, what percentage of these groups really contribute to explaining costs). In general, it is assumed that larger number of cost groups produce more accurate cost information and, statistically, increasingly larger number of groups are expected to yield better values of the reduction in variance. However, Krabbe-alkemade et al. noticed that, in some cases, a more fine-grained cost accounting system may not produce more accurate cost information due to measurement or classification errors [8]. Furthermore, they observed that higher number of groups with high reduction in variance may reduce efficiency in the sense of including many groups that do not match actual cases and could be, therefore, considered unnecessary. This was the case of our analysis, in which a relatively small APR-DRG groups covered a very high percentage of costs, suggesting that the inclusion of that many groups does not add much benefit to the overall estimate. On the other hand, the distribution of D_groups in the Queralt system showed high proportionality respect the percentage of costs and, more notably, hospitalizations, suggesting higher efficiency.

### Numeric measures vs. groups

Regardless of the different efficiency and performance of risk groups of the compared tools, it is worth mentioning that the Queralt system can be used either as grouper or numerical index. This feature overcomes several drawbacks of forcing cases into groups, such as information loss, underestimation of the variation in outcome, and concealment of non-linearity between variables and outcomes [16]. The use of the numerical index resulted in a relatively good symmetry between the observed and estimated costs using linear models; in this analysis, the APR-DRG showed a discontinuous shape, probably due to the group-based approach.

### Predicting costs

The broad range and heterogeneity of hospitalizations precludes an exact prediction of costs and, therefore, certain degree of disparity between predicted and actual costs must be assumed. In this regard, it is particularly important that these tools have high capacity to predict the highest costs. In our analysis, all tools performed well in predicting hospital costs at the 80th, 90th, and 95th percentiles, with slightly better performance of the Queralt DP_Strata in the three scenarios. In the PR analysis, which reflects the level of false positive and false negative rates, the DP_Strata clearly outstood compared with all other models.

### Limitations

Our analysis has some limitations that need to be mentioned. First, as all retrospective analyses based on information stored in electronic health records, the accuracy of our prediction depends on the quality of the data. However, since we are comparing two models using the same dataset, inaccuracies regarding resource use and diagnoses is expected to affect the two tools similarly. Regarding the origin of data, it is also worth mentioning that the Queralt system was developed using data from the same population―albeit collected in 2018. Thus, although costs were not considered in its development, an external validation, replicating the analysis in another population would increase the strength of our conclusions and inform on the generalizability of the model.

### Concluding remarks

In summary, the Queralt system offers valuable tools not only for predicting hospital outcomes (as originally intended) but also for predicting hospitalization costs. The combination of diagnosis and procedures strata yields approximately 600 groups that accurately reflect resource utilization during hospital stay by considering the main diagnosis, patient complexity on admission, and procedures needed. The Queralt system also had higher efficiency, with better match with the case-mix of hospitalizations found in routine care. Although future studies assessing the performance of this tool in other countries are warranted, the Queralt system offer healthcare managers of systems with comprehensive collection of information regarding diagnoses and procedures with a free tool that accurately reflect hospital costs and can be tailored, if necessary, to the specific needs of each environment.

### Electronic supplementary material

Below is the link to the electronic supplementary material.

Supplementary Material 1

## Data Availability

The dataset contains financial data from the Catalan Institute of Health and, therefore, is not publicly available. The software and users’ manual for the Queralt system can be freely accessed from https://ics.gencat.cat/ca/assistencia/coneixement-assistencial/sistema-de-Queralt/.

## Abbreviations

APR-DRG: All-patient refined DRG
DRG: Diagnosis-related groups
ICS: Catalan Institute of Health
ICU: Intensive care unit
Queralt DxC: Queralt index for in-hospital complications
Queralt DxP: Queralt index for principal diagnosis
Queralt DxS: Queralt index for diagnoses on admission
Queralt Px: Queralt index for procedures
