# Supplementary material for: Retrospective analysis of hospitalization costs using two payment systems: the diagnosis related groups (DRG) and the Queralt system, a newly developed case-mix tool for hospitalized patients
Source: Health Econ Rev. 2024 Jun 26;14:45. doi: 10.1186/s13561-024-00522-6 (PMC11202329; doi:10.1186/s13561-024-00522-6)
Supplement: Supplementary file 1 — Supplementary Material 1 [file 13561_2024_522_MOESM1_ESM.pdf]

Retrospective analysis of hospitalization costs using two payment systems: the Diagnosis Related Groups (DRG) and the Queralt system, a newly developed case-mix tool for hospitalized patients

Supplementary appendix

Supplementary Methods

D_strata and P_strata have been formulated using the following algorithm:

1. The indicators are sorted using their natural order, based on seriousness. For each of them, i, let CRF_i be its corresponding cumulative relative frequency and I_{max} the number of indicators.
2. Let N_MAX be the maximum number of desired groups.
3. Each indicator i, will be assigned to the greatest G such that:
    a. $G = \frac{r}{N\_MAX}, \ r \ \leq \ I_{max}$
    b. $CRF_i \geq G$

For the D_strata and P_strata, we have used N_MAX = 25, obtaining 21 and 17 groups respectively.

The DP_strata have been obtained by applying the Cartesian product between D_strata and P_strata.

Supplementary Figures and Tables

Figure S1. Distribution of costs (**A**) and hospitalizations (**B**) within each of the 20 most common DRG indexes and, within them, its severity. Colors show the relative values for the severity, from yellow (lowest values) to dark blue (highest values).

A

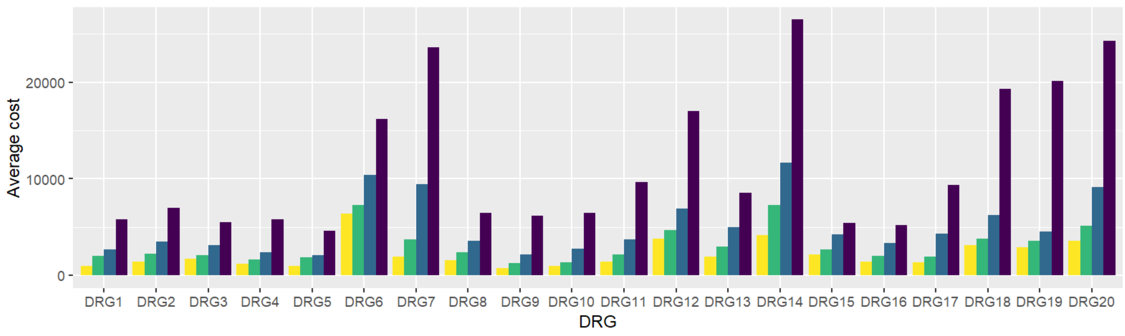

B

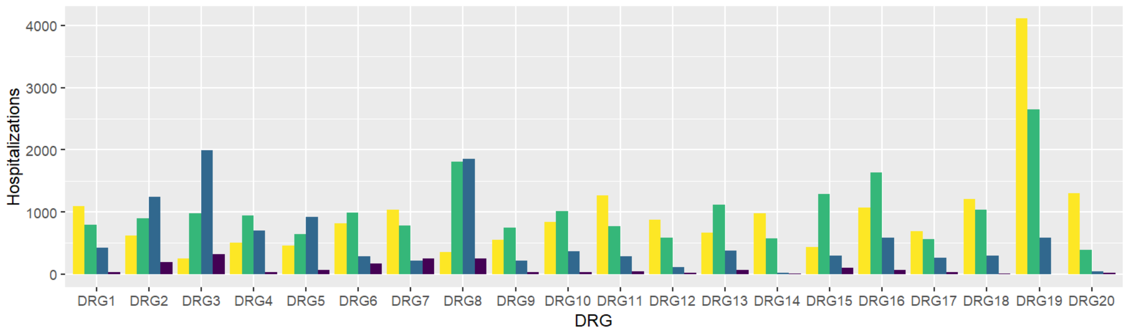

Figure S2. Mean cost according to age and sex (purple women, yellow men) for the eight more frequent hospitalization causes.

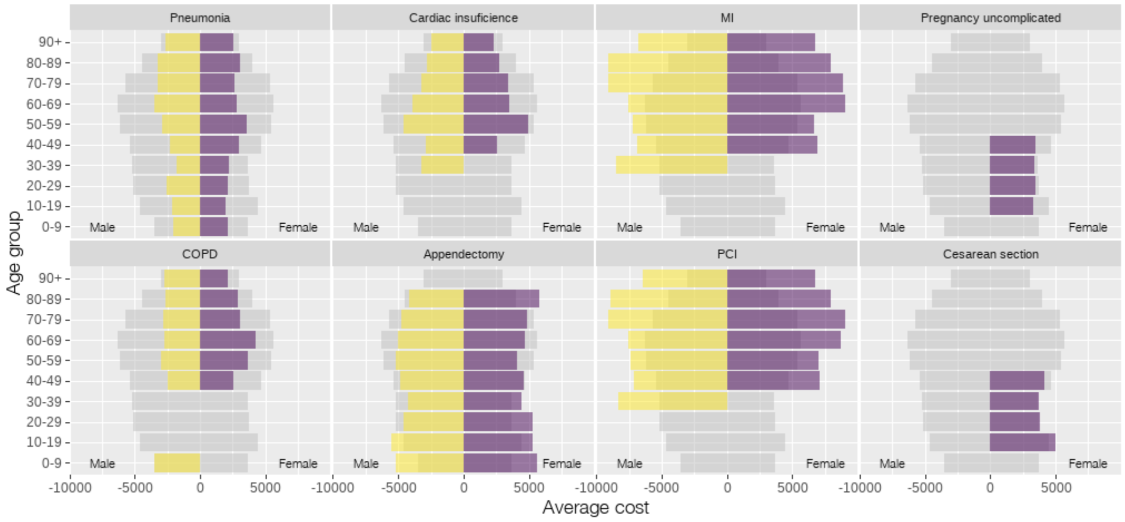

The Queralt and DRG codes for the selected diagnoses/procedures are as follows:

	Queralt Dx	DRG	Figure S1 Ref
Heart failure	CIR019	194	DRG8
Chronic obstructive pulmonary disease	RSP008	140	DRG3
Myocardial infarction	CIR009	174	DRG6
Pneumonia	RSP002	139	DRG2
Uncomplicated pregnancy	PRG029	560	DRG19
	**Queralt Px**		
Percutaneous interventions	CAR004	174	DRG6
Apendicectomy	GIS008	234	NA
Cesarea incision	PGN003	540	DRG18

Table S1. Cost (in €) of hospitalization episodes according to combinations of age and sex.

	Age group	Mean	Median	25th percentile	75th percentile
Sex					
Male	0-9	3514.6	1676.0	874.0	3223.8
Male	10-19	4610.0	2266.5	1050.8	4509.3
Male	20-29	5152.5	2412.0	891.0	5309.5
Male	30-39	5194.2	2557.0	860.0	5389.5
Male	40-49	5377.2	2700.0	1029.3	6053.3
Male	50-59	6157.6	3160.5	1199.8	6997.5
Male	60-69	6307.4	3251.5	1207.0	7272.5
Male	70-79	5707.0	3025.0	1113.0	6678.0
Male	80-89	4464.9	2487.0	841.0	5292.8
Male	90-99	3025.5	1789.0	634.0	3906.3
Male	100+	2189.7	967.0	574.0	2867.0
Female	0-9	3684.4	1616.0	888.0	3184.0
Female	10-19	4453.7	2071.0	873.0	3817.0
Female	20-29	3713.6	2521.5	1438.3	3833.8
Female	30-39	3645.7	2546.0	1738.0	3918.0
Female	40-49	4685.5	2788.0	1134.0	5433.0
Female	50-59	5411.4	3111.0	1155.0	6259.5
Female	60-69	5641.0	3233.0	1238.0	6583.0
Female	70-79	5369.3	3044.5	1015.8	6550.0
Female	80-89	3973.4	2254.0	705.0	5031.0
Female	90-99	2994.5	1761.0	607.0	4060.0
Female	100+	2951.7	1698.0	525.5	4126.8

Table S2. Area under the Receiving Operation Characteristics (ROC) and precision-recall (PR) curves.

	Percentiles		
	**95th**	**90th**	**80th**
	**AUC ROC**		
**APR-DRG**	0.5216	0.604	0.6985
**Queralt DP**	0.748	0.7965	0.834
**Queralt P**	0.6624	0.6811	0.7528
**Queralt D**	0.5748	0.639	0.7225
	**AU PRC**		
**APR-DRG**	0.9038	0.8817	0.8627
**Queralt DP**	0.958	0.9454	0.9277
**Queralt P**	0.9237	0.9063	0.8876
**Queralt D**	0.9177	0.8963	0.8769
